# Hydrogen peroxide‐mediated conversion of coproheme to heme *b* by HemQ—lessons from the first crystal structure and kinetic studies

**DOI:** 10.1111/febs.13930

**Published:** 2016-11-14

**Authors:** Stefan Hofbauer, Georg Mlynek, Lisa Milazzo, Dominic Pühringer, Daniel Maresch, Irene Schaffner, Paul G. Furtmüller, Giulietta Smulevich, Kristina Djinović‐Carugo, Christian Obinger

**Affiliations:** ^1^ Department for Structural and Computational BiologyMax F. Perutz LaboratoriesUniversity of ViennaAustria; ^2^ Dipartimento di Chimica ‘Ugo Schiff’Università di FirenzeSesto Fiorentino (FI)Italy; ^3^ Division of BiochemistryDepartment of ChemistryBOKU – University of Natural Resources and Life SciencesViennaAustria; ^4^ Department of BiochemistryFaculty of Chemistry and Chemical TechnologyUniversity of LjubljanaSlovenia

**Keywords:** coproheme, Gram‐positive pathogens, heme *b*, heme biosynthesis, HemQ

## Abstract

Heme biosynthesis in Gram‐positive bacteria follows a recently described coproporphyrin‐dependent pathway with HemQ catalyzing the decarboxylation of coproheme to heme *b*. Here we present the first crystal structure of a HemQ (homopentameric coproheme‐HemQ from *Listeria monocytogenes*) at 1.69 Å resolution and the conversion of coproheme to heme *b* followed by UV‐vis and resonance Raman spectroscopy as well as mass spectrometry. The ferric five‐coordinated coproheme iron of HemQ is weakly bound by a neutral proximal histidine H174. In the crystal structure of the resting state, the distal Q187 (conserved in Firmicutes HemQ) is H‐bonded with propionate p2 and the hydrophobic distal cavity lacks solvent water molecules. Two H_2_O_2_ molecules are shown to be necessary for decarboxylation of the propionates p2 and p4, thereby forming the corresponding vinyl groups of heme *b*. The overall reaction is relatively slow (*k*_cat_/*K*_M_ = 1.8 × 10^2^ m ^−1^·s^−1^ at pH 7.0) and occurs in a stepwise manner with a three‐propionate intermediate. We present the noncovalent interactions between coproheme and the protein and propose a two‐step reaction mechanism. Furthermore, the structure of coproheme‐HemQ is compared to that of the phylogenetically related heme *b*‐containing chlorite dismutases.

**Database:**

Structural data are available in the PDB under the accession number 5LOQ.

**Abbreviations**
5c: five‐coordinated
6c: six‐coordinated
AoCld: chlorite dismutase from *Azospira oryzae*
cld: chlorite dismutase
coproheme: coproporphyrin III
DaCld: chlorite dismutase from *Dechloromonas aromatica*
DyP: dye‐decolorizing peroxidase
ECD: electronic circular dichroism
FMN: flavin mononucleotide
HS: high‐spin
LmHemQ: HemQ from *Listeria monocytogenes*
LS: low‐spin
MaCld: chlorite dismutase from *Magnetospirillum* sp.
MS: mass spectrometry
NdCld: chlorite dismutase from ‘*Candidatus* Nitrospira defluvii’
NwCld: chlorite dismutase from *Nitrobacter winogradskyi*
p2: propionate in coproheme at position 2
p4: propionate in coproheme at position 4
p6: propionate in coproheme and heme *b* at position 6
p7: propionate in coproheme and heme *b* at position 7
RR: resonance Raman
SaHemQ: HemQ from *Staphylococcus aureus*

## Introduction

Heme biosynthesis in monoderm (Gram‐positive) bacteria follows a novel coproporphyrin‐dependent pathway described recently by Dailey and coworkers 1. The missing link in understanding this noncanonical bacterial heme biosynthesis pathway was HemQ which was shown to catalyze the decarboxylation of two propionate groups at positions 2 (p2) and 4 (p4) of coproporphyrin III (coproheme), thereby producing protoporphyrin IX (heme *b*) 2. Coproheme seems to act as both substrate and redox‐active cofactor as its conversion is triggered by the addition of FMN, hydrogen peroxide, or peracetic acid 1, 3, 4. However, no structural data of coproheme‐HemQ are available and the mechanism of the two decarboxylation steps is unknown.

Many Firmicutes and Actinobacteria are pathogens and a major threat to public health, due to the development of multiple resistance to common antibiotics 5, 6. Their steady‐state iron uptake, their heme biosynthesis, heme degradation, and iron release is crucial for the viability of these bacterial organisms and depend strongly on HemQ 7, the latter might be a promising therapeutic target in the future. However, this requires a detailed knowledge of structure–function relationships that at present is lacking; only apo‐structures of HemQs are available in the Protein Data Bank (www.pdb.org) 8. The interaction of apo‐HemQ from *Listeria monocytogenes* with coproheme was previously evaluated by means of molecular dynamics simulations of a coproheme‐bound model of LmHemQ, based on the apo‐structure (4WWS), suggesting hydrogen bonds between Y147 and S225 and p4 as well as between Q187 and p2 9. Coproheme binding to apo‐HemQ was shown to be biphasic resulting in the formation of mainly high‐spin ferric protein with a standard reduction potential of the Fe(III)/Fe(II) couple of −205 mV at pH 7.0 9. Celis *et al*. and Lobo *et al*. reported that ferric coproheme‐HemQ needs an excess of hydrogen peroxide to mediate the two decarboxylation steps of coproheme to heme *b* and that under such conditions, heme bleaching and destruction occurred 3, 4. Additionally, it was demonstrated that the conversion proceeds stepwise yielding an intermediate three‐propionate monovinyl, monopropionate deuteroheme isomer III 3.

Herein we report the first coproheme‐bound HemQ structure at 1.69 Å resolution, which gives valuable information about the interaction of the substrate and redox‐cofactor coproheme with the active site, including coproheme coordination and noncovalent interactions of the four propionates with the protein matrix. Comparison between apo‐LmHemQ and coproheme‐LmHemQ structures (HemQ from *L. monocytogenes*) allows identification of the amino acid residues likely involved in catalysis as well as in a gating function in the course of substrate binding. Additionally, by UV‐vis and electronic circular dichroism (ECD) spectroscopy as well as mass spectrometry, we demonstrate a clear two‐step kinetics of the conversion of coproheme to heme *b* as well as a H_2_O_2_ to coproheme ratio of 2 : 1. The presence of excess of H_2_O_2_ leads to the irreversible formation of heme‐protein linkages and heme bleaching. The data will be discussed with regard to the proposed physiological role of HemQ in heme biosynthesis of Gram‐positive bacteria and possible reaction mechanism(s). In addition, they are compared to the phylogenetically related chlorite dismutases (Clds) and dye‐decolorizing peroxidases (DyPs) 10, 11, 12 which share a similar overall fold but exhibit completely different enzymatic properties and display distinct differences in the architecture and amino acid composition of the proximal and distal sides of the heme cavity 13.

## Results

### Crystal structure of coproheme‐LmHemQ

In this work, we heterologously expressed and purified apo‐HemQ from *L. monocytogenes* (LmHemQ) as described previously 8. The enzyme was reconstituted by the addition of equimolar concentrations of coproheme. Coproheme‐LmHemQ (7.5 mg·mL^−1^) was crystallized in 0.1 m bicine pH 9.0, 10% w/v PEG6000, and diffracted to a resolution of 1.69 Å. Crystals were pale red.

Coproheme‐LmHemQ crystallized as a pentamer in space group P12_1_1, with unit cell dimensions of 77.69 Å × 129.37 Å × 77.91 Å. The final model (PDB code: 5LOQ) consists of 9801 protein atoms, 713 water molecules, has good stereochemistry, and *R*‐factors (Table 1). The Wilson temperature factor is 26.8 Å^2^. A clear electron density was observed for residues 4–251 of subunit A; for subunit B, 6–111 and 125–251; for subunit C, from 3 to 113 and 126 to 251; for subunit D, from 2 to 251; and for subunit E, from 6 to 113 and 125 to 251. The missing amino acids are part of a loop from S111 to N123. The weak electron density in this region indicates flexibility of this loop. In chain A and D, the loop is fully resolved due to crystal packing contacts.

**Table 1:** Data collection and refinement statistics

	Coproheme‐LmHemQ (5LOQ)
Data collection	
Wavelength (nm)	0.9537
Resolution range (Å)	47.08–1.69 (1.75–1.69)^a^
Space group	P12_1_1
Unit cell length *a*,* b*,* c* (Å)	77.69, 129.4, 77.92
E.S.D. of *a*,* b*,* c* (Å)	5.5E‐02, 1.0E‐01, 5.5E‐02
Unit cell angle α, β, γ (°)	90, 105.52, 90
E.S.D. of α, β, γ (°)	0, 2.3E‐02, 0
Total reflections	471 367 (46 069)
Unique reflections	162 948 (16 108)
Multiplicity	2.9 (2.9)
Completeness (%)	0.98 (0.98)
Mean *I*/sigma (*I*)	2.93 (0.39)
Wilson B‐factor (Å^2^)	26.8
Refinement	
*R*‐merge (%)	18.9 (174.8)
*R*‐meas (%)	23.1 (214.1)
*R*‐pim (%)	13.1 (122.3)
CC1/2	0.96 (0.174)
CC*	0.99 (0.545)
Reflections used in refinement	161 813 (15 055)
Reflections used for *R*‐free	1976 (185)
*R*‐work (%)	18.4 (40.7)
*R*‐free (%)	21.5 (41.9)
CC (work)	0.957 (0.511)
CC (free)	0.962 (0.643)
Number of nonhydrogen atoms	10 796
No. of atoms macromolecules	9801
No. of atoms ligands	282
No. of atoms solvent	713
Protein residues	1203
RMS (bonds) (Å)	0.011
RMS (angles) (°)	1.12
Ramachandran favored (%)	99
Ramachandran allowed (%)	1.3
Ramachandran outliers (%)	0.084
Rotamer outliers (%)	2.4
Clashscore	1.68
Average B‐factor (Å^2^)	38.5
Macromolecules (Å^2^)	37.5
Ligands (Å^2^)	68.4
Solvent (Å^2^)	41.5
Number of TLS groups	50

a Values in parentheses are for the highest resolution shell.

**Figure 1:**
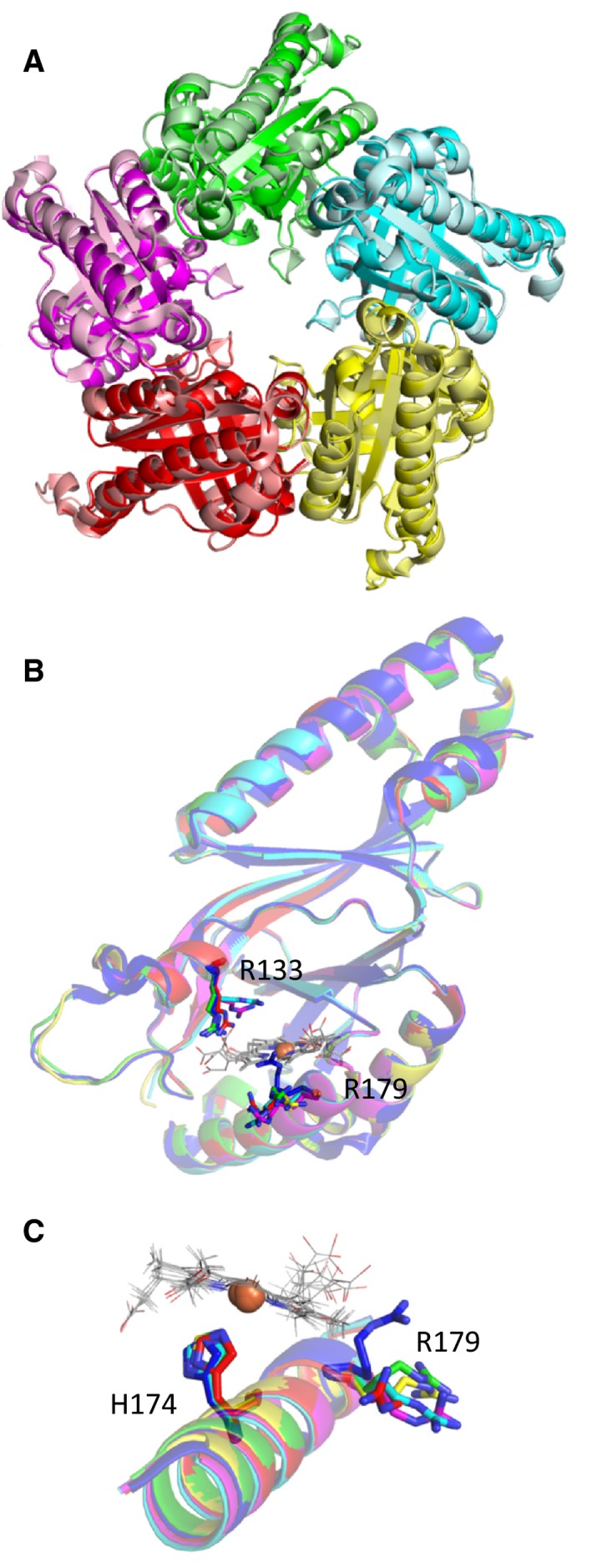
Structure of coproheme‐LmHemQ. (A) Overlay of pentameric apo‐LmHemQ (4WWS, pale colors) and with pentameric coproheme‐HemLmQ (5LOQ, saturated colors) (B) Overlay of apo‐LmHemQ (4WWS: chainA) shown as a blue cartoon with all five subunits of coproheme‐LmHemQ (chain A: green, chain B: pink, chain C: cyan, chain D: yellow, chain E: red). R133 and R179 for apo‐LmHemQ are shown as blue sticks and for coproheme‐LmHemQ subunits as lines in the respective colors. Coprohemes are depicted in light gray. (C) Overlay as in (B) of the helix on the proximal side of the coproheme, where the proximal histidine (H174) and R179 are located.

Apo‐LmHemQ (PDB code: 4WWS) crystallizes in the same oligomeric state and space group with similar unit cell dimensions (Fig. 1A). Subunit A of apo‐LmHemQ 8 and coproheme‐LmHemQ (with flexible loop S111‐N123 resolved in both structures) superimpose with a root‐mean‐square deviation (RMSD) of 0.223 Å over 225 Cα atoms. A noteworthy difference between the structures of apo‐LmHemQ and coproheme‐LmHemQ concerns the orientation of two arginine side chains (R133, R179) at the substrate access channel. Arginine 133 of apo‐LmHemQ and coprohemeQ exhibit the same conformation in chain A but different conformations in the other subunits. The flexibility of this side chain is also supported by weak electron densities. R179 shows different conformations in the five subunits and thus no distinct electron density can be assigned (due to crystal contacts, only R179 of chain B shows a distinct electron density) (Fig. 1B,C).

Additionally, the oligomeric assembly of coproheme‐LmHemQ shows further structural heterogeneities of heme cavity residues and the substrate/cofactor within the five subunits. All coprohemes are anchored by a proximal histidine residue (H174) with coproheme iron distances between 2.4 and 3.0 Å. Comparison with the structures of the phylogenetically related wild‐type and mutant chlorite dismutases (Clds) shows that the distances between the *N*_ε_ of the imidazole of the proximal His and the iron ion are shorter (Table 2). Both the longer bond length and the high variability of distances in coproheme‐HemQ suggest that the proximal histidine is not part of a hydrogen bond network as seen in the related Clds and DyPs. Note that the α‐helix (M164‐Y181) harbors both H174 as well as the (flexible) R179 residue (Fig. 1C).

**Table 2:** Distances between the heme iron and the *N*_ε_ of the imidazole of the proximal histidine in all available chlorite dismutase structures (www.pdb.org)

PDB code	Protein	Ligand	Resolution	pH	Minimum distance (Å)	Maximum distance (Å)	Average distance (Å)	Subunits per asymmetric unit	Reference
5LOQ	Copro‐LmHemQ	–	1.69	9.0	2.4	3.0	2.64 ± 0.21	5	This work
3NN1	NdCld wild‐type	Imidazole	1.85	7.5	2.1	2.1	2.10	5	30
3NN2	NdCld wild‐type	Cyanide	1.94	7.5	2.2	2.2	2.20	5	30
3NN3	NdCld R173A	–	2.60	4.6	2.2	2.3	2.24 ± 0.05	5	30
3NN4	NdCld R173K	–	2.70	4.0	2.1	2.2	2.18 ± 0.04	5	30
4M05	NdCld R173E	–	2.28	4.5	2.1	2.2	2.16 ± 0.05	5	31
4M06	NdCld W145F	Cyanide	2.60	4.5	2.1	2.2	2.12 ± 0.04	5	31
4M07	NdCld W145F		2.50	4.5	2.1	2.3	2.20 ± 0.06	5	31
4M08	NdCld W145V	Imidazole	2.80	4.0	2.3	2.5	2.44 ± 0.08	5	31
4M09	NdCld W146Y R173Q	Imidazole	2.45	3.5	2.5	2.6	2.58 ± 0.04	5	31
2VXH	AoCld	Thiocyanate	2.10	5.5	2.1	2.2	2.13 ± 0.05	6	32
3Q08	DaCld	Nitrite	3.05	6.5	2.2	2.6	2.33 ± 0.10	20	33
3Q09	DaCld	Nitrite	3.00	9.0	2.0	2.3	2.14 ± 0.08	20	33
5A12	MaCld	Azide	1.40	6.0	2.1	2.2	2.16 ± 0.05	5	34
5A13	MaCld	Thiocyanate	1.75	8.5	2.1	2.2	2.17 ± 0.05	10	34
3QPI	NwCld	–	2.10	7.9	2.1	2.2	2.15 ± 0.05	2	35

**Figure 2:**
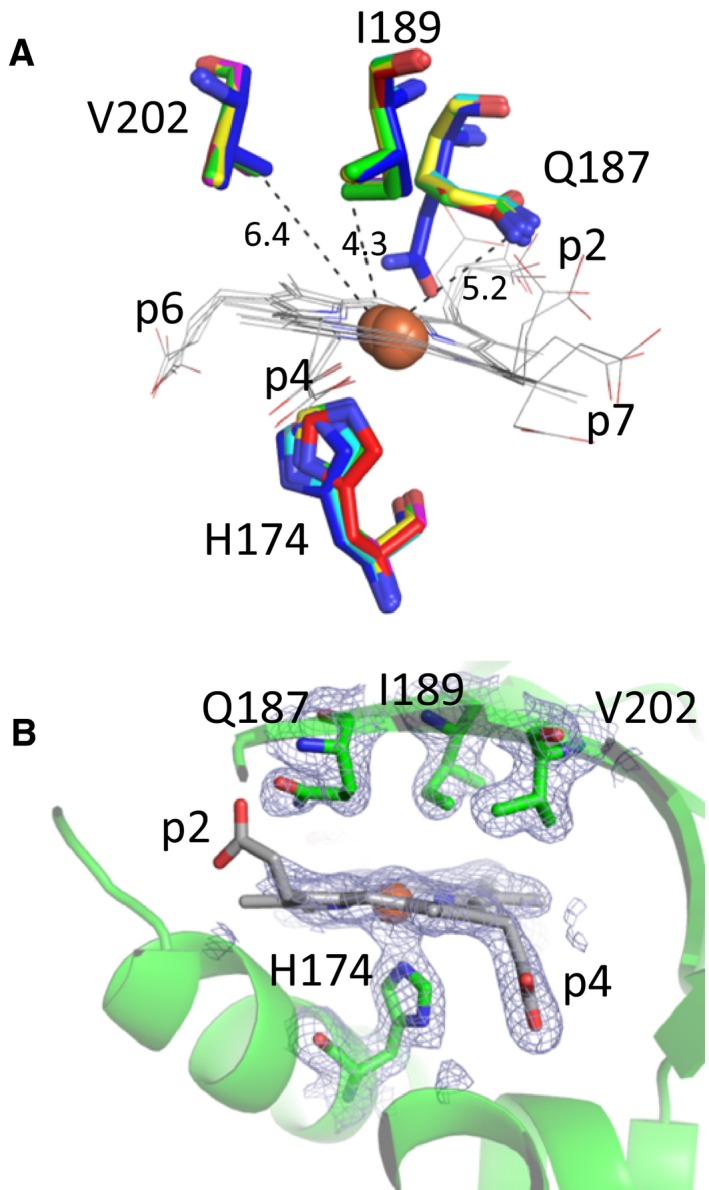
Active site architecture of coproheme‐LmHemQ. (A) Distal residues and proximal histidine (H174) in all five subunits are depicted as green, pink, cyan, yellow, and red sticks. Residues of apo‐LmHemQ structure (4WWS) are presented in blue. Coproheme of all subunits is depicted as gray lines. (B) Distal and proximal residues of coproheme in chain A are represented as green sticks within the cartoon representation of the secondary structure. Electron density map (2mFo‐DFc) contoured at 1 σ is depicted as blue mesh.

The amino acids of the distal side of the coproheme‐LmHemQ complex are Q187 (which aligns with the catalytic arginine of chlorite dismutases), I189, and V202; distances to the heme iron are 5.2 Å (Q187), 4.3 Å (I189), and 6.4 Å (V202). Q187 is oriented toward the protein surface, pointing away from the coproheme iron in coproheme‐LmHemQ (Fig. 2A). Interestingly, no electron densities of potential solvent molecules are present on the distal side (Fig. 2B).

**Figure 3:**
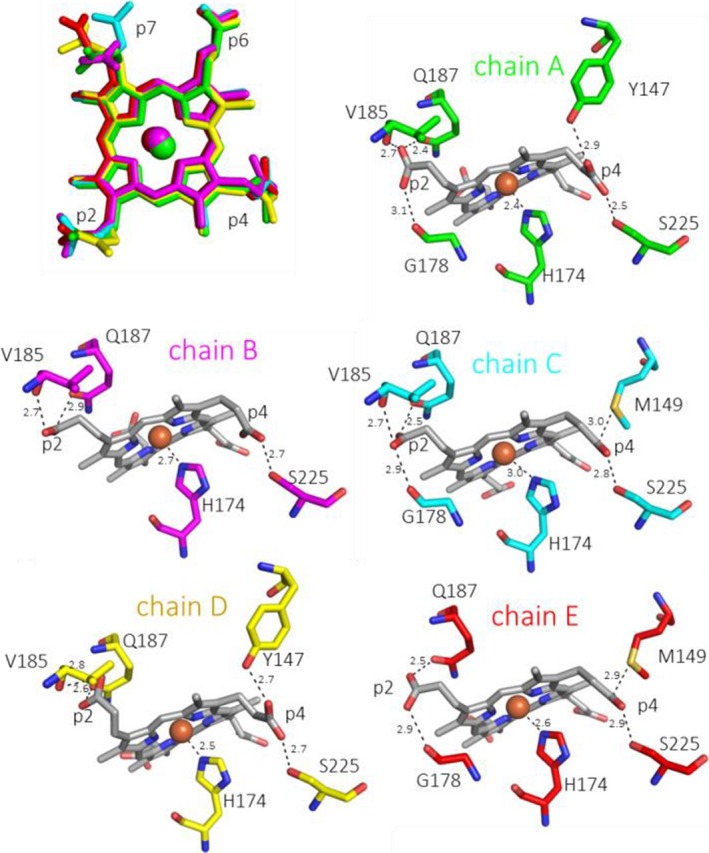
Coproheme conformation and environment. Overlay of coproheme moieties bound to LmHemQ from all five subunits (top left), and H‐bonding interactions of coproheme propionates at positions 2 and 4 with the protein matrix in the respective subunits, residues within 3 Å of a propionate oxygen atom are depicted as sticks. The coordination of the proximal H174 to the coproheme iron is also shown.

Comparing the coprohemes of all five subunits clearly demonstrates higher flexibility of those propionates that are closer to the protein surface, namely p2 and p7. The conformational freedom of p4 and p6 is much more restricted (Fig. 3). Note that upon conversion of coproheme to heme *b*, p2 and p4 are eliminated. H‐bond analyses of the catalytically relevant p2 and p4 clearly identify Y147, M149, S225 as H‐bonding partners for p4, and G178, V185, Q187 as H‐bonding partners for p2 (Fig. 3). The interactions of Y147 and S225 with p4 and Q187 with p2 were previously correctly predicted *in silico* using MD simulations 9.

**Figure 4:**
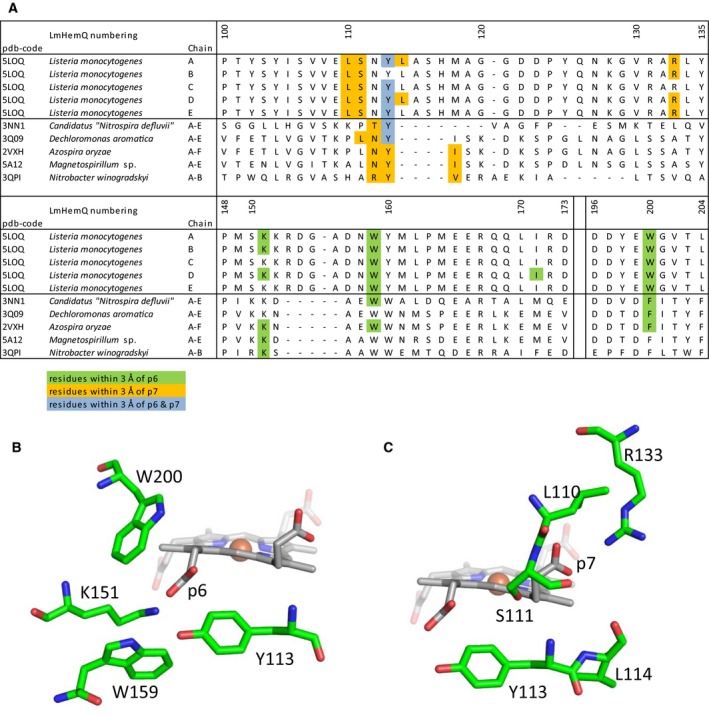
Structural environment of p6 and p7. (A) Sequence alignment of LmHemQ and chlorite dismutases with available crystal structures. Residues with atoms within 3 Å of the propionate oxygen atoms of p6 are highlighted in green, of p7 in orange, and if both propionates are within the threshold it is highlighted in blue. The alignment was generated previously 9. Stick presentation of residues within 3 Å of p6 (B) and p7 (C) of chain A of coproheme‐LmHemQ.

The interaction of p6 and p7 (which are not cleaved off by HemQ and therefore are also present in the heme *b* of structurally related chlorite dismutases) with the protein moiety is shown in Fig. 4A, where residues within a distance of 3 Å to p6 or p7 are highlighted, not necessarily building H‐bonds with the propionates (Fig. 4B,C). In LmHemQ and Clds, conserved residues interact with p6 and p7 (Y113 for p7; K151, W159 for p6), and also residues unique for HemQ are in close proximity to the two propionates. The most prominent one is R133, which potentially forms H‐bonds with p7 and is located at the substrate entrance channel; this arginine aligns with alanine, leucine, or valine of Clds.

As described above, in the structure of coproheme‐LmHemQ, only in chain A and chain D, a flexible loop between L114 and Y125 could be completely built, because of reliable electron densities due to crystal packing contacts. In the apo‐structure of LmHemQ (PDB code: 4WWS) 8, the loop was only resolved in chain A and E, exhibiting the same conformation as in coproheme‐LmHemQ, probably because this conformation was stabilized by the crystal packing contacts. MD simulations confirmed the flexibility of this loop 9. Interestingly, this loop is only present in HemQs from Firmicutes and not from Actinobacteria or in Cld/HemQ‐like proteins from Archaea (alignment shows the absence of 10 residues) 14.

### UV‐vis and resonance Raman spectroscopy of ferric and ferrous coproheme‐LmHemQ and heme *b*‐LmHemQ

**Figure 5:**
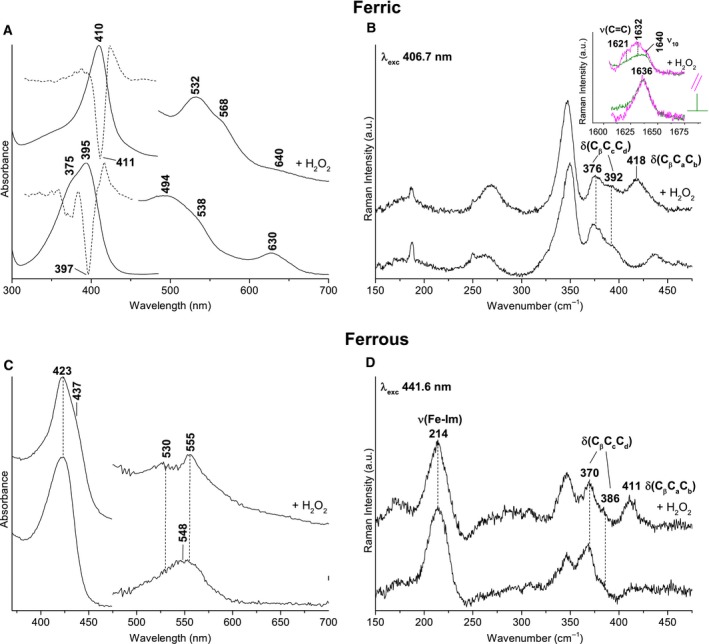
UV‐vis and resonance Raman spectra of ferric and ferrous forms of coproheme‐LmHemQ and heme *b*‐LmHemQ in 50 mm phosphate buffer, pH 7.0. (A) UV‐vis and second derivative spectra of ferric coproheme‐LmHemQ and heme *b*‐HemQ. Heme *b*‐LmHemQ was formed by using a 5 : 1 stoichiometric excess of H_2_O_2_ to coproheme‐LmHemQ. (B) Resonance Raman spectra of ferric coproheme‐LmHemQ and heme *b*‐LmHemQ in the low frequency region showing the propionyl and vinyl bending modes. The inset shows the vinyl stretching modes region in polarized light. Experimental conditions: excitation wavelength 406.7 nm, laser power at the sample 5 mW; average of six spectra with 60‐min integration time (coproheme‐LmHemQ), average of four spectra with 40‐min integration time (heme *b*‐LmHemQ). (C) UV‐vis spectra of ferrous coproheme‐LmHemQ and heme *b*‐HemQ (D) Resonance Raman spectra of ferrous coproheme‐LmHemQ and heme *b*‐LmHemQ in the low frequency region showing the ν(Fe‐Im) stretching mode together with the propionyl and vinyl bending modes. Experimental conditions: excitation wavelength 441.6 nm, laser power at the sample 10 mW; average of six spectra (coproheme‐LmHemQ) and of three spectra (heme*b*‐LmHemQ) with 30‐min integration time.

The spectroscopic signatures of LmHemQ are comparable to those reported for HemQ from *Staphylococcus aureus* (SaHemQ) 3. The UV‐vis spectrum of ferric coproheme‐LmHemQ shows a Soret band at 395 nm (shoulder at 375 nm) and a very strong CT1 band at 630 nm typical of high‐spin (HS) heme (Fig. 5A). Considering the overall blue‐shift of the spectrum compared to that of other heme proteins due to the lack of vinyl conjugation, the electronic absorption spectrum appears typically five‐coordinated (5c). The UV‐vis spectrum of ferric heme *b*‐LmHemQ (produced by addition of hydrogen peroxide to coproheme‐HemQ) shows the Soret maximum at 410 nm and a weak CT1 at 640 nm, typical of a six‐coordinated low‐spin (6cLS) heme with a minor HS component (Fig. 5A). Comparison of the low frequency region of the RR spectra of coproheme‐LmHemQ and heme *b*‐LmHemQ did not show any significant intensity change in the propionyl bending modes, assigned to the broad bands at 376 and 392 cm^−1^ (Fig. 5B). Also in case of HemQ from *S. aureus* (SaHemQ) 3, changes in intensity of the propionyl bending modes (at 378 and 390 cm^−1^) are evident only by inspection of the difference spectra between heme *b*‐LmHemQ and coproheme‐LmHemQ. By contrast, the formation of the vinyl substituents can be clearly observed. The spectra in polarized light allowed us to assign the ν(C=C) stretching modes (polarized) at 1621 and 1632 cm^−1^ (Fig. 4B, inset). Moreover, in the low frequency region, the δ(C_β_C_a_C_b_) vinyl modes are observed as a single band at 418 cm^−1^ (Fig. 5B), similar to that observed at 416 cm^−1^ for SaHemQ 3.

The UV‐vis spectrum of ferrous coproheme‐LmHemQ is characteristic of a predominant 5cHS species in the absence of vinyl conjugation (Soret at 423 nm and maximum at 548 nm), whereas ferrous heme *b*‐LmHemQ is a mixture of 6cLS (Soret at 423 nm) and 5cHS (Soret at 437 nm) species (Fig. 5C). Upon conversion of coproheme‐LmHemQ to heme *b*‐LmHemQ, two new bands at 530 and 555 nm appear (at the expense of the band at 548 nm), consistent with the significantly increased amount of LS species. For both proteins, the ν(Fe‐Im) stretching mode is observed at 214 cm^−1^ (Fig. 5D). This is identical to that reported for SaHemQ 3, suggesting that the *N*_ε_ of the imidazole of the proximal His (H174) is weakly bonded to the heme iron in agreement with the crystal structure and the interatomic distances (2.4–3.0 Å). This finding clearly demonstrates that conversion of p2 and p4 to the respective vinyl groups has no impact on the proximal heme coordination and architecture.

### Kinetics of conversion of coproheme to heme *b*

**Figure 6:**
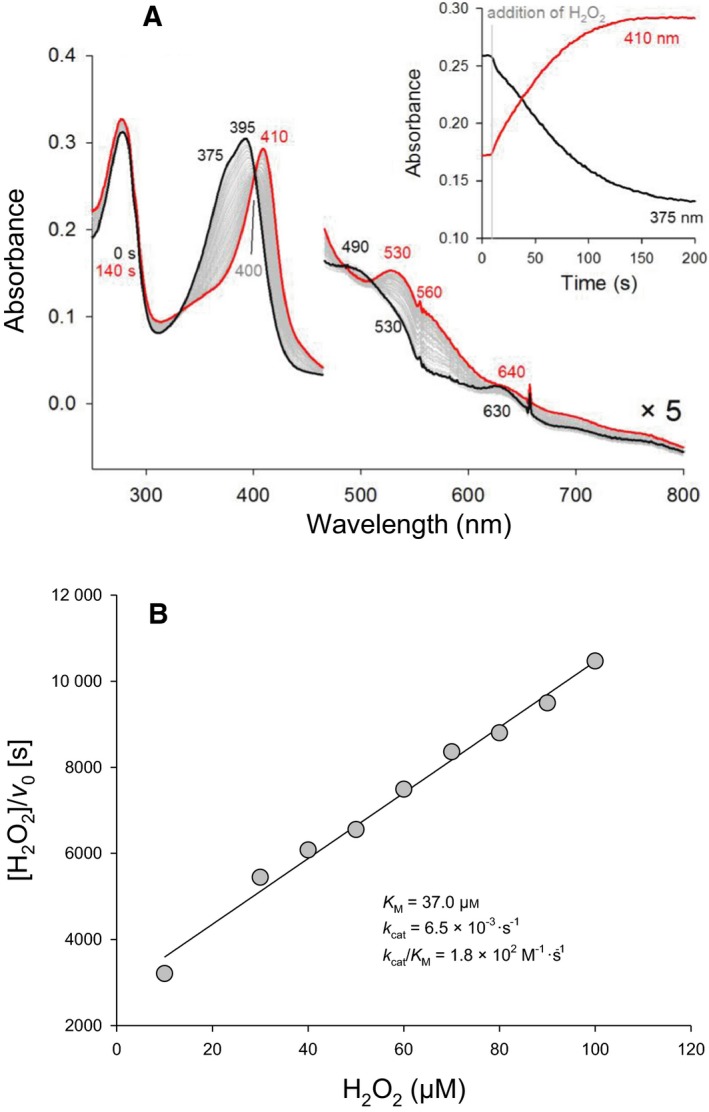
Enzymatic activity of coproheme‐LmHemQ mediated by H_2_O_2_. (A) Spectral conversion of 5 μm coproheme‐LmHemQ mediated by addition of 50 μm H_2_O_2_. The initial coproheme‐LmHemQ spectrum is depicted in black, the resulting final heme *b*‐LmHemQ spectrum in red. Selected spectra during conversion are shown in gray. The inset depicts time traces, extracted at 370 and 410 nm. (B) Kinetics of the H_2_O_2_‐mediated conversion of coproheme‐LmHemQ in 50 mm phosphate buffer, pH 7.0, are presented as Hanes plot.

Upon addition of hydrogen peroxide to coproheme‐LmHemQ, decarboxylation of the propionate substituents of coproheme leads to the formation of heme *b‐*LmHemQ. During this reaction, the original spectrum of coproheme‐LmHemQ (Soret maximum: 395 nm) converts to a spectrum with the characteristics of heme *b‐*HemQ (Soret maximum: 410 nm) with an isosbestic point at 400 nm (Fig. 6A) 3, 8. When a 10‐fold excess of H_2_O_2_ is used (5 μm coproheme‐HemQ, 50 μm H_2_O_2_), the reaction is completed after 140 s (Fig. 6A, inset).

To obtain catalytic parameters for this H_2_O_2_‐mediated reaction, initial velocities (*v*_0_) were obtained by monitoring the absorbance increase at 410 nm. The observed kinetic behavior is shown in Fig. 6 and the apparent *K*_M_ value for H_2_O_2_ was calculated to be 37.1 μm (Fig. 6B). The turnover number is very low (~ 0.0065 s^−1^) resulting in a *k*_cat_/*K*_M_ value of approximately 1.8 × 10^2^ m ^−1^·s^−1^ (Fig. 6B) at pH 7.0. In contrast to SaHemQ, heme bleaching is not observed within the investigated initial reaction phase (~ 50 s) (Fig. 6A) 3. Coproheme concentration could not be varied, as free coproheme (if present in excess) is bleached by H_2_O_2_.

### Stoichiometry of the H_2_O_2_‐driven decarboxylation reactions

**Figure 7:**
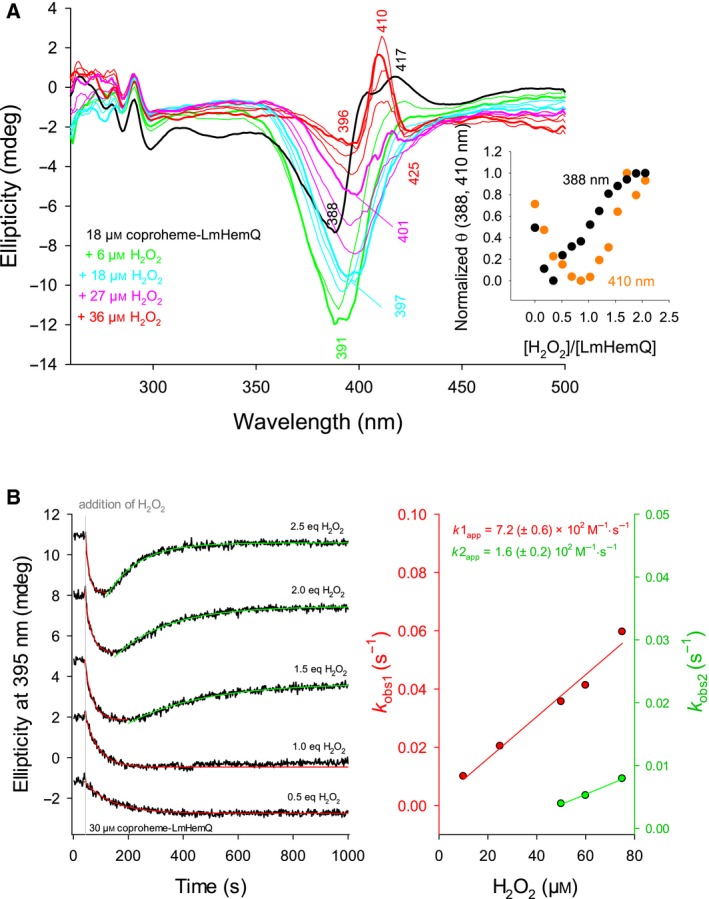
Reaction of H_2_O_2_‐mediated coproheme conversion to heme *b* followed by electronic circular dichroism. (A) Spectral conversion of 18 μm coproheme‐LmHemQ mediated by stepwise titration with H_2_O_2_ (0–36 μm). Spectrum of coproheme‐LmHemQ is depicted as bold black line (0 μm H_2_O_2_), final spectrum as bold red line (36 μm H_2_O_2_). Bold green line, bold cyan line, bold pink line represent addition of 6, 18, and 27 μm H_2_O_2_, respectively. Thin lines in the respective color are at H_2_O_2_ concentrations lower than the one represented by a bold line (green: 3 μm; cyan: 9 μm, 15 μm; pink: 21 μm, 24 μm; red: 30 μm, 33 μm H_2_O_2_). The inset depicts the normalized changes in ellipticity at 388 (black) and 410 nm (orange) after each titration step versus the H_2_O_2_/coproheme‐LmHemQ ratio. (B, left panel) Time traces (black lines) of conversion of 30 μm coproheme‐LmHemQ to heme *b*‐LmHemQ followed by the change of ellipticity at 395 nm. Single exponential fits are depicted in red (first phase) and green (second phase). The time of H_2_O_2_ addition is indicated as gray line, spectra were shifted for clarity. (B, right panel) Plots of *k*_obs1_ (red) and *k*_obs2_ (green) versus H_2_O_2_ concentration with linear fits. Conditions: 50 mm phosphate buffer, pH 7.0.

**Figure 8:**
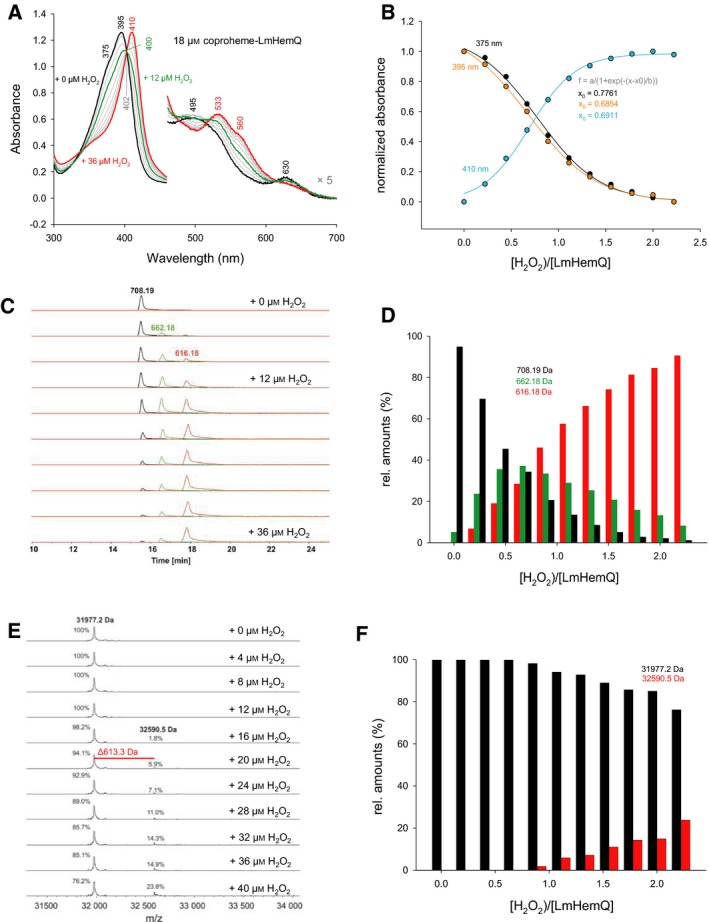
Stoichiometry of the enzymatic activity of coproheme‐LmHemQ mediated by H_2_O_2_. (A) UV‐vis absorption spectra recorded following the stepwise titration of 18 μm coproheme‐LmHemQ with H_2_O_2_ (0–36 μm). (B) Plot of normalized absorbance changes at 375 (black), 395 (orange), and 410 nm (cyan) after each titration step versus the H_2_O_2_/coproheme‐LmHemQ ratio (including sigmoidal fits). (C) HPLC profiles and mass spectrometric analysis of samples from the titration experiment described in (A); coproheme (708.19 Da, black), monovinyl, monopropionate deuteroheme (662.18 Da, green), heme *b* (616.18 Da, red). (D) Relative amounts of the three porphyrin species (area under curve of HPLC profiles) at each H_2_O_2_/coproheme‐LmHemQ ratio. (E) Whole protein analyses of the samples from the titration experiment described in (A) and (C). (F) Relative amounts of LmHemQ without cross‐linked heme (black, 31 977.2 Da) and cross‐linked heme (red, 32 590.5) depending on the [H_2_O_2_]/coproheme‐HemQ ratio. Conditions: 50 mm phosphate buffer, pH 7.0.

The absence of heme bleaching allowed the determination of the H_2_O_2_ to coproheme stoichiometry for this reaction. Coproheme‐LmHemQ was titrated with subequimolar concentrations of H_2_O_2_ followed by ECD spectroscopy (Fig. 7A), UV‐vis spectroscopy (Fig. 8A,B), and mass spectrometry (Fig. 8C,D).

The ECD spectrum of coproheme‐LmHemQ exhibits a minimum at 388 nm and a maximum at around 417 nm (Fig. 7A, black line). Upon reaction with 0.3 eq H_2_O_2_, the ellipticity at 417 nm decreases and a prominent minimum at 391 nm is formed (Fig. 7A, bold green line). At increasing hydrogen peroxide, the ellipticity of this minimum continuously decreases and red‐shifts. At 2 eq H_2_O_2_, the final spectrum with a maximum at 410 nm is observed (i.e., heme *b*‐HemQ) (Fig. 7A, bold red line). This spectral conversion together with the absence of isosbestic points clearly suggest the presence of at least one intermediate species with ECD features different from coproheme‐HemQ and heme *b*‐HemQ. This is also evident from plotting normalized ellipticities at 388 and 410 nm against the stoichiometric ratio of H_2_O_2_ over LmHemQ (Fig. 7A, inset). The ellipticities initially decreased and subsequently increased again showing turning points just below 0.5 equivalents (388 nm) and 1.0 equivalents (410 nm) of H_2_O_2_ to coproheme‐LmHemQ. The reaction was completed after reaching a twofold stoichiometric excess of hydrogen peroxide.

The stepwise conversion of coproheme to heme *b* is also supported by following the change in ellipticity at 395 nm with time (Fig. 7B). Time traces exhibit a clear biphasic behavior, where the first (single exponential) phase represents the formation of the intermediate and the second, slower (single exponential) phase represents the formation of the product heme *b*. Apparent bimolecular rates for the first and the second transitions were approximated to be 7.2 ± 0.6 × 10^2^ m ^−1^·s^−1^ and 1.6 ± 0.2 × 10^2^ m ^−1^·s^−1^, respectively (Fig. 7B). These values are in good agreement with the determined steady‐state parameter *k*_cat_/*K*_M_ of approximately 1.8 × 10^2^ m ^−1^·s^−1^ at pH 7.0 (Fig. 6B) for the overall reaction.

Next, the stoichiometry of the reaction was followed by UV‐vis spectroscopy (Fig. 8A). The normalized absorbances at 375 and 395 nm (Soret maximum of coproheme‐HemQ, shoulder at 375 nm) as well as 410 nm (Soret maximum of heme *b*‐HemQ) from these studies were plotted against the stoichiometric ratio of H_2_O_2_ over LmHemQ. Fitting with a sigmoidal function reveals midpoints of conversion at [H_2_O_2_]/LmHemQ ratios between 0.6854 and 0.7761. The reaction is complete after reaching a twofold stoichiometric excess of H_2_O_2_ (Fig. 8B).

Finally, during the UV‐vis titration, small aliquots were taken for mass spectrometric analysis at each H_2_O_2_ concentration. The corresponding HPLC elution profiles of coproheme (708.19 Da), monovinyl, monopropionate deuteroheme (662.18 Da), and heme *b* (616.18 Da) are presented in Fig. 8C. Integration of the area under the respective peaks reveals that the initial sample (black spectrum in Fig. 8A, top HPLC profile in Fig. 6C) contains almost exclusively coproheme (~ 95%). At 12 μm H_2_O_2_ (ratio of 0.66), coproheme, monovinyl, monopropionate deuteroheme, and heme *b* are present in fairly equal amounts (coproheme: 34%; monovinyl, monopropionate deuteroheme: 37%; heme *b*: 29%), whereas after addition of 36 μm H_2_O_2_ (ratio of 2.0), heme *b* is the most abundant species detected (~ 85%) (Fig. 8D). This clearly demonstrates that the reaction occurs in a stepwise manner with a three‐propionate species as intermediate. However, the mass spectrometric data did not allow to distinguish between monovinyl, monopropionate deuteroheme isomer III or IV (same molar mass).

Mass spectrometric analysis of the titration of coproheme‐LmHemQ with H_2_O_2_ also included whole protein analysis of the samples. The obtained data clearly showed cross‐linking of the heme group with the protein, starting at approximately equimolar H_2_O_2_ concentrations (Fig. 8E,F). After addition of 40 μm H_2_O_2_ to 18 μm coproheme‐LmHemQ, 23.8% of the protein was covalently cross‐linked to a modified prosthetic group (Fig. 8E,F).

## Discussion

It has been recently discovered that HemQ 2 is a key element in the heme biosynthetic pathway of monoderm bacteria 1. Coproheme was identified as the substrate which is decarboxylated at p2 and p4 to form heme *b*. While the physiological role of HemQ now seems to be elucidated (although several other roles have been proposed), the knowledge regarding structure–function relationships was limited until now. The catalytic efficiency of the proposed reaction is rather low but this is reasonable in a biological context. The apparent *k*_cat_/*K*_M_ value for HemQ from *L. monocytogenes* for H_2_O_2_ was determined to be 1.8 × 10^2^ m ^−1^·s^−1^, very similar to that reported for HemQ from *S. aureus* (SaHemQ) 3. *In vivo* in the Gram‐positive bacterial cell, H_2_O_2_ most likely is not present in (high) micromolar concentrations, but is continuously provided by HemY and consumed by coproheme‐HemQ 1, 14. This consequently means that the conditions used to determine Michaelis–Menten parameters (Fig. 6) do not reflect *in vivo* conditions. This assumption is also supported by the fact that coproheme‐HemQ is very susceptible to irreversible inhibition by modification of the substrate/cofactor and the protein in the presence of a stoichiometric excess of H_2_O_2_.

Consequently, the H_2_O_2_‐mediated decarboxylation reaction was studied under subequimolar concentrations of H_2_O_2_ to minimize side reactions. In this way, it was possible to demonstrate that complete conversion of 1 mol coproheme to 1 mol heme *b* requires 2 mol of hydrogen peroxide. The reaction occurs in a stepwise manner with the formation of a three‐propionate intermediate (Figs 7 and 8) and needs Fe‐coproheme for catalysis, as Ni‐coproheme‐HemQ displays no enzymatic activity 1. This observation suggests that coproheme acts as both substrate and redox‐cofactor. Furthermore, it suggests that hydrogen peroxide mediates the oxidation of ferric coproheme‐HemQ to a reactive (electron‐deficient) compound I‐like state, as observed in heme peroxidases and catalases [i.e., oxoiron(IV) in combination with either a porphyryl radical (Por^•^^+^), (i.e., Compound I) or with an amino acid radical (aa^•^^+^), (i.e., Compound I*]. This redox intermediate initiates a radical mechanism which restores the Fe(III) state and converts the propionyl to the vinyl substituent thereby releasing carbon dioxide. To affect the decarboxylation of p2 and p4, two reaction cycles and thus two H_2_O_2_ molecules are necessary. The detection of the three‐propionate intermediate, as well as the spectral changes observed during turnover by ECD spectroscopy (which show biphasic behavior, including a turning point in ellipticity) suggest that these two decarboxylation steps occur consecutively (Figs 7 and 8).

Both the UV‐vis and RR spectra as well as the standard reduction potential of the Fe(III)/Fe(II) couple of coproheme‐LmHemQ of −205 mV 9 demonstrate that the enzyme is in the ferric state under physiological conditions. The crystal structure of coproheme‐LmHemQ together with the RR data suggest a relatively weak interaction between the heme iron and *N*_ε_ of the proximal ligand H174. Due to the absence of an H‐bonding partner, the anionic character of H174 must be significantly less pronounced compared to the phylogenetically related Clds. Another important difference between Cld and coproheme‐HemQ concerns the coordination of the heme iron at the distal side. Interestingly, the crystal structure of coproheme‐LmHemQ (in contrast to Clds) shows no electron densities of potential solvent molecules, which is also reflected by temperature‐dependent investigations of the standard reduction potential of the Fe(III)/Fe(II) couple of coproheme‐SaHemQ 9. This study revealed a highly negative Δ*S*°′_rc,solv_ value, which indicates a reduction‐induced increase in solvent ordering, which is typical for a system with a buried metal site and limited solvent accessibility to the distal heme cavity 9. By contrast, in Clds reduction of the ferric to the ferrous state is entropically favored 15. Limited accessibility is also reflected by cyanide binding rates that are two orders of magnitude slower compared to Clds 9, and it is reasonable to assume that this restricted access to the distal heme cavity concerns also the substrate hydrogen peroxide.

How H_2_O_2_ oxidizes coproheme‐HemQ for production of a redox intermediate that initiates the oxidative decarboxylation reaction is unknown. In contrast to typical heme peroxidases of the peroxidase‐catalase and peroxidase‐cyclooxygenase superfamilies 10 and catalases 16, which typically provide a distal His‐Arg or His‐Asn pair for heterolytic cleavage of H_2_O_2_ and Compound I formation, in coproheme‐HemQ comparable catalytic residues are absent. The distal amino acids within 6 Å distance from the heme iron are Q187, I189, and V202, respectively. Glutamine 187, which aligns with the catalytic arginine in Clds, is fully conserved in HemQs from Firmicutes but is exchanged by alanine in (putative) HemQs from Actinobacteria. In the crystal structure of coproheme‐LmHemQ, Q187 is in the ‘out’ conformation (distance between Fe and amide nitrogen: 5.2 Å) making a hydrogen bond with the carboxyl group of p2. According to MD simulations 9, this H‐bond is formed during ~ 80% of the 30 ns simulation time 9. By contrast in the structure of apo‐LmHemQ, Q187 is in the ‘in conformation’ (distance between Fe and amide nitrogen: 3.5 Å). This suggests that in heme *b*‐LmHemQ, Q187 the ‘in’ conformation is possible (due to the absence of p2) which might contribute to the very pronounced LS character of heme *b*‐LmHemQ compared to coproheme‐LmHemQ.

The hydrophobic nature of the distal heme cavity, the absence of a catalytic base for deprotonation of H_2_O_2_, the absence of distal water molecules, and thus of an obvious pathway for proton delivery or elimination, suggest that formation of the initial reaction product [i.e., enzyme‐peroxide complex, Fe(III)‐O‐O‐H] must be extremely slow compared to heme peroxidases or heme catalases and could be the rate‐limiting step of the enzyme turnover. For the subsequent redox reaction, the cleavage of the peroxide bond either follows a heterolytic mechanism [Fe(III)‐O‐O‐H → Fe(IV) = O Por^•^^+^ + H_2_O] or a homolytic cleavage. However, in HemQ, the absence of (a) a distal His‐Arg pair, which supports heterolytic cleavage by acting as proton donating and polarizing catalytic amino acids, and (b) of an anionic proximal histidine, which also promotes heterolytic bond cleavage, suggest a homolytic mechanism [Fe(III)‐O‐O‐H → Fe(IV) = O + ^•^OH]. The hydroxyl radical then could attack either p2 or p4 to form water and the corresponding propionyl radical (p˙). Celis *et al*. 3 have proposed an alternative homolytic pathway which generates Fe(IV) = O, H_2_O, and p^•^ in a concerted manner without releasing the ^•^OH radical.

In the classic heme biosynthesis pathway of diderm (Gram‐negative) bacteria, HemN decarboxylates coproporphyrinogen III (no iron inocorporated) to protoporphyrinogen IX. It utilizes a [4Fe‐4S] cluster cofactor and SAM‐dependent mechanism to cleave off propionates at positions 2 and 4 17. In this reaction, SAM is cleaved to methionine and a highly oxidizing 5′‐deoxyadenosyl radical, which subsequently abstracts a hydrogen atom from the β‐C atom of the propionate side chain. The formation of the propionyl radical initiates the decarboxylation reaction and, finally, after a second one‐electron oxidation step, the vinyl group is formed 17. Except for the formation of the propionyl radical, coproheme‐HemQ could follow the same mechanism. The conversion of 1 mol coproheme to 1 mol heme *b* starts with the oxidation of the resting state by H_2_O_2_. Compound I*, [Fe(IV) = O CoproPor‐propionate^•^) is built by one of the two homolytic pathways described above (Reaction 1). Finally, by intramolecular transfer of the unpaired electron to the iron and decarboxylation, the ferric state is restored and the vinyl group is formed (Reaction 2). For total conversion of coproheme [Fe(III) coproPor‐propionate] to heme *b* [Fe(III) Por‐vinyl], two redox cycles have to be passed.

$$\left[\text{Fe}\left(\text{III}\right)\text{coproPor‐propionate}\right]+H_{2}O_{2}\to \left[\text{Fe}\left(\text{IV}\right)=O\;{\text{coproPor‐propionate}}^{∙}\right]+H_{2}O\left(\text{Reaction}\;1\right)$$

$$\left[\text{Fe}\left(\text{IV}\right)=O\;{\text{coproPor‐propionate}}^{∙}\right]\to \left[\text{Fe}\left(\text{III}\right)\text{Por‐vinyl}\right]+{\text{CO}}_{2}+H_{2}O\left(\text{Reaction}\;2\right)$$

The involvement of a (reactive) Compound I‐like intermediate in this reaction is also supported by the fact that in the presence of an excess of H_2_O_2_ (after complete conversion of p2 and p4 to the respective vinyl substituents) formation of a crosslink between the cofactor (i.e., reaction product heme *b*) and the protein occurs (Fig. 8E,F). Formation of covalent heme‐protein bonds is known from mammalian 18 and bacterial heme peroxidases 19, 20 and typically follows an autocatalytic radical mechanism. This observation is most probably an *in vitro* artifact and not relevant *in vivo*, where heme *b* is easily released and taken up by an apo‐protein 9. Nevertheless, it underlines that the oxidized state of cofactor‐bound HemQ is highly reactive.

The open question remains why p2 and p4 (and not p6 and p7) of coproheme are decarboxylated. The first crystal structure of coproheme‐HemQ of *L. monocytogenes* now allows to critically investigate the environment of these substituents. Interestingly, both p6 and p7 form H‐bonds with the positively charged amino acids K151 and R133 (Fig. 4), which impairs the formation of propionyl radicals. By contrast, p2 interacts with (distal side) Q187 (conserved in all Firmicutes, but is exchanged by alanine in Actinobacteria), V185 (mostly conserved in both phylae), and G178 (conserved in Firmicutes, but exchanged by alanine in Actinobacteria) 9. Residues interacting with p4 are Y147, M149, and S225, with Y147 being fully conserved in all Firmicutes and Actinobacteria. Methionine 149 is present in Firmicutes and aligns with phenylalanine in Actinobacteria. Serine 225 is mostly conserved in Firmicutes, but interestingly is exchanged in HemQ from *S. aureus* by a threonine. In Actinobacteria, an arginine is found at the respective position 9. The impact of these amino acid residues (e.g., Q187, Y147, and M149) on Reactions 1 and 2 as well as the observed differences between HemQs in Firmicutes and Actinobacteria must be investigated in the future. To date, a HemQ from Actinobacteria has not been investigated on a protein level.

In conclusion, HemQ is a homopentameric enzyme that uses coproheme as substrate and redox‐cofactor for the conversion of coproheme to heme *b*. For the relatively slow and stepwise conversion of coproheme to heme *b*, two H_2_O_2_ molecules are necessary. The heme cavity architecture together with biochemical/physical data suggest homolytic cleavage of hydrogen peroxide and a radical mechanism that includes two one‐electron oxidations of the propionate substituent (p2 and p4). As a consequence of interaction with basic amino acids, the propionates p6 and p7 seem to be protected from oxidation.

## Experimental procedures

### Expression and purification of LmHemQ

LmHemQ was subcloned into a modified version of the pET21(+) expression vector with an N‐terminal StrepII‐tag, cleavable TEV protease, expressed in *Escherichia coli* Tuner (DE3) cells (Merck/Novagen) and purified via a StrepTrap HP 5 mL column (GE Healthcare) as described in previously 8. The enzyme was reconstituted by the addition of equimolar concentrations of free Fe(III) coproporphyrin III chloride (coproheme; Frontiers Scientific, Logan, UT, USA) (in 50 mm phosphate buffer, pH 7.0, predissolved in 0.5 m NaOH) to apo‐LmHemQ dissolved in 50 mm phosphate buffer, pH 7.0. In order to determine the extinction coefficient of coproheme, various concentrations of coproheme solutions in 50 mm phosphate buffer, pH 7.0, were prepared and UV‐vis absorption spectra were recorded with an Agilent 8453 diode array spectrophotometer (Hewlett‐Packard, Palo Alto, CA, USA). The absorbance at 390 nm was plotted against the coproheme concentrations and from the slope, the extinction coefficient for coproheme at 390 nm was obtained (128 800 m ^−1^·cm^−1^).

### Enzymatic activity of LmHemQ

The conversion of coproheme to heme *b* by LmHemQ was followed spectroscopically at 410 nm (ε_410_ = 76 600 m ^−1^·cm^−1^) 9. Solution of 2 μm coproheme‐LmHemQ in 50 mm phosphate buffer, pH 7.0, was used and the reactions were started by the addition of 10–100 μm H_2_O_2_ (Sigma, Vienna, Austria). The H_2_O_2_ concentration was checked spectrophotometrically prior to each measurement (ε_240_ = 39.4 m ^−1^·cm^−1^) 21. The initial linear phase was used to determine the Michaelis–Menten parameter. Production rates of heme *b* (μm s^−1^) were plotted against H_2_O_2_ concentrations, and *K*_M_ and *v*_max_ were calculated using a Hanes plot [extraction of catalytic parameters from the slope (1/*v*_max_) and the intercept (*K*_M_/*v*_max_)].

The activity of LmHemQ was followed spectroscopically between 250 and 700 nm using the cyclic measurement mode (every 2.2 s, a spectrum was recorded for 200 s, Specord 10 photodiodearray photometer from Zeiss, Jena, Germany). In this experiment, 5–50 μm H_2_O_2_ was added to 5 μm coproheme‐LmHemQ, in 50 mm phosphate buffer, pH 7.0.

### Reaction of H_2_O_2_ with coproheme‐LmHemQ followed by electronic circular dichroism spectroscopy

Electronic circular dichroism spectroscopy was performed using Chirascan (Applied Photophysics, Leatherhead, UK). The instrument was flushed with a nitrogen flow of 5 L·min^−1^ and allowed simultaneous monitoring of UV‐vis electronic absorption and circular dichroism. Coproheme‐LmHemQ was analyzed in the near UV and visible region (260–500 nm; 5 s nm^−1^ scan speed, 1 nm bandwidth, 10 mm path length, stirred cuvette). Temperature was controlled by a Peltier element and kept at 25 °C. All measurements were performed in 50 mm phosphate buffer, pH 7.0. During titrations, subequimolar aliquots of H_2_O_2_ were added to the enzyme solution under constant stirring.

### Determination of the reaction stoichiometry—mass spectrometry

In order to determine the stoichiometry of H_2_O_2_ to coproheme, titrations were performed, where small (subequimolar) aliquots of H_2_O_2_ were added to coproheme‐LmHemQ. In a typical experiment, H_2_O_2_ was added in 2 μm aliquots to 18 μm coproheme‐LmHemQ. To ensure complete reaction of the H_2_O_2_, an interval of at least 15 min was set between each peroxide addition. The titrations were performed in a stirred cuvette (1 mL sample volume) at room temperature and monitored by UV‐vis absorption spectroscopy between 250 and 700 nm, using a scanning photometer (Hitachi U‐3900, Tokyo, Japan). About 20 μL of the sample was taken after each addition of H_2_O_2_ and analyzed by mass spectrometry.

For mass spectrometric analysis, 5 μL of each sample was analyzed using a Dionex Ultimate 3000 system directly linked to a QTOF mass spectrometer (maXis 4G ETD; Bruker, Rheinstetten, Germany) equipped with the standard ESI source of the positive ion mode. MS scans were recorded within a range from 300 to 3750 *m*/*z* and the instrument was tuned to detect both the rather small, free heme derivatives and intact proteins in a single run. Instrument calibration was performed using ESI calibration mixture (Agilent, Santa Clara, CA, USA). For separation of the analytes, a Thermo ProSwift™ RP‐4H Analytical separation column (250 × 0.2 mm) was used. A gradient from 99% solvent A and 1% solvent B (solvent A: 0.05% TFA, B: 80% ACCN and 20% solvent A) to 65% B in 11 min was applied, followed by a 2‐min gradient from 65% B to 95% B, at a flow rate of 8 μL min^−1^ and at 75 °C. A blank run (5 μL H_2_O) was performed after each sample to minimize carryover effects.

### Crystallization and structure determination

#### Crystallization

Crystallization experiments were performed using SWISSCI MRC three‐well crystallization plates (Molecular Dimensions, Newmarket, UK) adopting the vapor diffusion method. Crystallization drops were set using a Phoenix HT crystallization robot (Art Robins Instruments, Sunnyvale, CA, USA). The reservoir was filled with 40 μL crystallant solution. In the sample wells, ratios of 150 : 200 nL, 200 : 200 nL, and 250 : 200 nL protein (7.5 mg·mL^−1^) to crystallant were dispensed. Commercially available crystallization screens were used for initial screening. Crystallization plates were stored at 22 °C. Successful trials were obtained using the JCSG‐plus™ from Molecular Dimensions and SaltRx Screen from Hampton Research. Diffracting crystals grew in condition E10 of the JCSG‐plus™ screen (0.1 m bicine pH 9.0, 10% w/v PEG6000).

#### Harvesting and X‐ray data collection, structure determination, and refinement

Prior to data collection, the crystal was soaked with mother liquor supplemented with 25% MPD, harvested using cryo‐loops, and flash‐vitrified in liquid nitrogen. Datasets were collected at beam‐line ID29 22 of European Synchrotron Radiation Facility (ESRF, Grenoble, France) at 100 K using a DECTRIS PILATUS 6M detector.

The dataset was processed with xds and symmetry equivalent reflections merged with xdsconv 23. Intensities were not converted to amplitudes. The high‐resolution cutoff was based on a CC1/2* criterion 24. The phase problem was solved by molecular replacement using Phaser‐MR 25 taking PDB structure 4WWS, apo‐HemQ from *L. monocytogenes* 8. The model was further improved by iterative cycles of manual model building using coot 26 and maximum likelihood refinement using PHENIX‐Refine 27. PHENIX‐refine converted intensities into amplitudes using the French and Wilson algorithm 28. Restraints for coproheme (Ligand ID RM9) were generated using eLBOW taking a sdf file as input and applying the final‐geometry option. Final stages of refinement included Translation Liberation Screw (TLS) parameters, isotropic B‐factor model, automated addition of hydrogens and water molecules, optimization of X‐ray/ADP weight, and optimization of X‐ray/stereochemistry weight. The model was validated with MolProbity 29. Figures were prepared with pymol (http://www.pymol.org).

### Resonance Raman spectroscopy

#### Sample preparation

Ferric coproheme‐LmHemQ samples were prepared by adding free coproheme (in 50 mm phosphate buffer, pH 7.0, predissolved in 0.5 m NaOH) to apo‐LmHemQ dissolved in 50 mm phosphate buffer, pH 7.0. Ferric heme *b*‐LmHemQ samples were prepared by adding small aliquots of a concentrated solution of H_2_O_2_ in 50 mm phosphate buffer, pH 7.0 to coproheme‐LmHemQ. Ferrous samples were prepared by addition of a freshly prepared sodium dithionite (10 mg·mL^−1^) solution to the ferric forms previously degassed with nitrogen.

Sample concentrations were in the range 10–20 μm for both coproheme‐LmHemQ and heme *b*‐LmHemQ. The coproheme‐LmHemQ and heme *b*‐LmHemQ concentrations were determined using an extinction coefficient (ε) of 68 000 m ^−1^·cm^−1^ at 395 nm and 76 600 m ^−1^·cm^−1^ at 410 nm, respectively 9.

#### Electronic absorption measurements

Electronic absorption measurements were recorded using a 5 mm NMR tube (300 nm·min^−1^ scan rate) or a 1 cm cuvette (600 nm·min^−1^ scan rate) at 25 °C by means of a Cary 60 spectrophotometer (Agilent Technologies) with a resolution of 1.5 nm. Absorption spectra were measured both prior and after RR measurements to ensure that no degradation occurred under the experimental conditions used.

#### Resonance Raman measurements

The resonance Raman (RR) spectra were obtained at 25 °C using a 5 mm NMR tube by excitation with the 406.7 nm line of a Kr^+^ laser (Coherent, Innova 300 C; Coherent, Santa Clara, CA, USA) and with the 441.6 nm line of a He–Cd laser (Kimmon IK4121R‐G). Backscattered light from a slowly rotating NMR tube was collected and focused into a triple spectrometer (consisting of two Acton Research SpectraPro 2300i instruments and a SpectraPro 2500i instrument in the final stage with gratings of 3600 and 1800 grooves·mm^−1^) working in the subtractive mode, equipped with a liquid nitrogen‐cooled CCD detector. A spectral resolution of 1 cm^−1^ was calculated theoretically on the basis of the optical properties of the spectrometer for the 3600 grating. The RR spectra were calibrated with indene, n‐pentane, and carbon tetrachloride as standards to an accuracy of 1 cm^−1^ for intense isolated bands. All RR measurements were repeated several times (about 20 different samples) under the same conditions to ensure reproducibility. No changes have been observed within 30 min of irradiation with 5 mW (ferric) and 10 mW (ferrous) for laser excitation in the Soret band. To improve the signal‐to‐noise ratio, a number of spectra were accumulated and summed only if no spectral differences were noted (see caption of Fig. 5). All spectra were baseline‐corrected.

## Author contributions

SH, DP, IS, GM performed crystallization experiments. DP, GM refined the structure. SH, IS performed and analyzed activity assays. DM performed mass spectrometry analysis. LM, GS performed and analyzed resonance Raman spectroscopy experiments. The study was designed by SH, GS, PGF, KDC, CO. The paper was written by SH, GM, GS, PGF, KDC, CO. All authors reviewed the results and approved the final version of the manuscript.

## Conflict of interest

The authors declare no competing financial interests.
